# Vulnerability of mixotrophic algae to nutrient pulses and UVR in an oligotrophic Southern and Northern Hemisphere lake

**DOI:** 10.1038/s41598-017-06279-9

**Published:** 2017-07-24

**Authors:** P. Carrillo, J. M. Medina-Sánchez, M. Villar-Argaiz, F. J. Bullejos, C. Durán, M. Bastidas-Navarro, M. S. Souza, E. G. Balseiro, B. E. Modenutti

**Affiliations:** 1 Instituto Universitario de Investigación del Agua, Universidad de Granada, C/ Ramón y Cajal, 4, 18071 Granada, Spain; 2 Departamento de Ecología, Facultad de Ciencias, Universidad de Granada, Campus Fuentenueva s/n, 18071 Granada, Spain; 3 Department of Biosciences, The Faculty of Mathematics and Natural Sciences, 0371 Oslo, Norway; 4 Laboratorio de Limnología, INIBIOMA (CONICET-UNComahue), Quintral 1250, Bariloche, Río Negro R8400 Argentina

## Abstract

Nutrient inputs and ultraviolet radiation (UVR) are global factors affecting the structure and functioning of aquatic ecosystems, particularly clear-water ecosystems. We performed experiments in two model lakes highly exposed to UVR fluxes in order to test the effect that future increases in mineral nutrients transported by dust aerosol might exert on primary producers depending on the likelihood of atmospheric inputs. Lake La Caldera (Northern Hemisphere) has been receiving recurrent dust inputs from the Sahara Desert while lake Los Cántaros (Southern Hemisphere) has been less affected by dust aerosol. UVR × Nutrient synergistically stimulated primary production (PP), chlorophyll *a* (Chl *a*), with a smaller increase in phytoplanktonic biomass in La Caldera, but not in Los Cántaros, where nutrient addition unmasked the UVR inhibitory effect on phytoplankton. The proportional decrease of mixotrophic nanoflagellates (MNFs) after the nutrient pulse (in Los Cántaros) and the long-term decline of MNFs in La Caldera associated with the increase in aerosol-dust intrusions from the Sahara during the last 40 years suggest that a future scenario of intensified aerosol events from desert and desertified areas would not only reduce functional diversity with the decline of MNFs, but would ultimately alter the C flux towards the grazing chain in oligotrophic ecosystems.

## Introduction

Single natural or anthropogenic components of global environmental change have been extensively studied, but growing evidence underlines that global change exerts its effect through interactions among multiple stressors, leading to cumulative and/or non-additive impacts on ecosystems^1–4^. Crucial issues currently facing ecologists involve quantifying, understanding, and predicting the interaction of multiple drivers in a scenario of global change^5, 6^. Complex interactive effects on species and ecosystems include (i) trade-off mechanisms^7^, (ii) stress-induced tolerance^8^ and (iii) differences in the threshold of intensity or environmental sensitivity among trophic levels^9–12^. Additionally, the complexity of the interactive effects stems from the fact that multiple stressors, acting in concert, can exacerbate or buffer the impact on organisms more than predicted from their individual effects, as one given stressor may intensify or counteract the impact of the other^13, 14^.

Because information on the interaction modes and target sites for many stressors is still controversial, future studies should focus on variables that, by representing key biological processes, help identify early ecosystem responses to multiple stressors^15^. Among these, primary production (PP) is a key process affected by global change both in aquatic and terrestrial ecosystems^16^. Because it affects the exchange of CO_2_ between the water and the lower atmosphere and influences the amount of energy and materials entering the ecosystems, alterations of PP provide a valuable tool to assess the vulnerability of aquatic ecosystems to global change^17–19^.

Relevant global factors affecting the C flux in ecosystems are, among others, ultraviolet radiation (UVR) and changes in nutrient inputs brought about by both climate change (e.g. atmospheric deposition, wind, precipitation) and increased human activity^20, 21^. In remote areas such as Alpine lakes, the main nutrient inputs are dust-aerosol transport by wind from source areas (e.g. a desert^22^). These events move great amounts of mineral nutrients (mainly phosphorus [P]^23^) and may cause stress in oligotrophic areas (*sensu* ^2^; see ref. 24). The largest dust emissions in the world come from North Africa (Sahara Desert) with significant regional effects in the northern temperate and equatorial areas^22^. Nevertheless, Brahney *et al*.^25^, recently combining observational data and an atmospheric-simulation model, have suggested that greater atmospheric P depositions could determine the nutrient state of Alpine lakes, especially in the Southern Hemisphere.

There is growing environmental awareness concerning the role of UVR in ecosystems in the current climate-change scenario^26^. Zonal average UV irradiance has become more intense in the Southern Hemisphere (at 50°S: 305 nm [UVB-radiation], 23%; erythemal [erythemal action spectrum weighted irradiances] 8.5%) in comparison to the Northern one (at 50°N: 305 nm, 9%; erythemal, 4%) from 1978 to 2008^27^. Although the Montreal Protocol was successful at banning ozone-depleting substances, recovery of this atmospheric layer is complex, as it depends on latitude and greenhouse-gas emissions^26^, and it is not expected to be complete until 2025–2040 at mid-latitudes^28^. UVR, therefore, still remains a world-wide stressor with far-reaching implications for the functioning of the aquatic community^18^. UVR is known to be responsible for multiple effects on primary producers, including DNA damage, inhibition of photosynthesis or changes in aquatic-community structure^18, 19^. In addition, UVR affects the ability of autotrophs to acquire nutrients inhibiting the P uptake or stimulating it^29, 30^. Thus, it is crucial to understand how UVR and nutrients interact on primary producers, because alterations in these organisms have a consequence for the C flux in the aquatic ecosystems^31^.

Although laboratory and field experiments have provided evidence for the strong interactive effects of UVR and nutrients on the aquatic community, results are still controversial. While, some studies have reported decreased algal sensitivity to UVR after P addition^32^, others have shown no influence^33^. Furthermore, it has been shown that the P-addition unmasks the deleterious effect of UV on primary producers in clear oligotrophic freshwater^24, 34–36^ and marine ecosystems^37^. These results open new questions concerning the joint role of UVR and nutrients in clear-water ecosystems that demand further research: Do ecosystems differ in their responses based on their likelihood to receive atmospheric inputs? What are the mechanistic bases for the generated responses? How might changes in climatic regimes (e.g. drought frequency) affect biodiversity in response to nutrient inputs to aquatic ecosystems?

To answer these questions, we firstly analysed long-term data series in Lake La Caldera (Sierra Nevada, Spain) to test whether natural aerosol events are proximate drivers of shifts in phytoplanktonic community composition. Secondly, we tested the hypothesis that nutrient pulses unmask the harmful effect of UVR on primary producers in oligotrophic highly UVR exposed lakes in both hemispheres. In addition, we anticipated less-intense effects in the response of primary producers to UVR and nutrient manipulations in the northern compared to the southern lake due to the higher frequency and magnitude of Saharan dust intrusions in the former lake.

Our hypothesis was tested through an experimental design performed in each lake, consisting of i) one-month treatments to evaluate the UVR effect in large mesocosms (allowing UVR-avoiding mechanisms to operate) followed by ii) mid-term UVR × Nutrient factorial design in microscosms mimicking current (in La Caldera lake, North Hemisphere) and potential (in Los Cántaros lake, South Hemisphere) nutrient inputs (Supplementary Fig. S1).

## Results

### Long-term data

**Figure 1:**
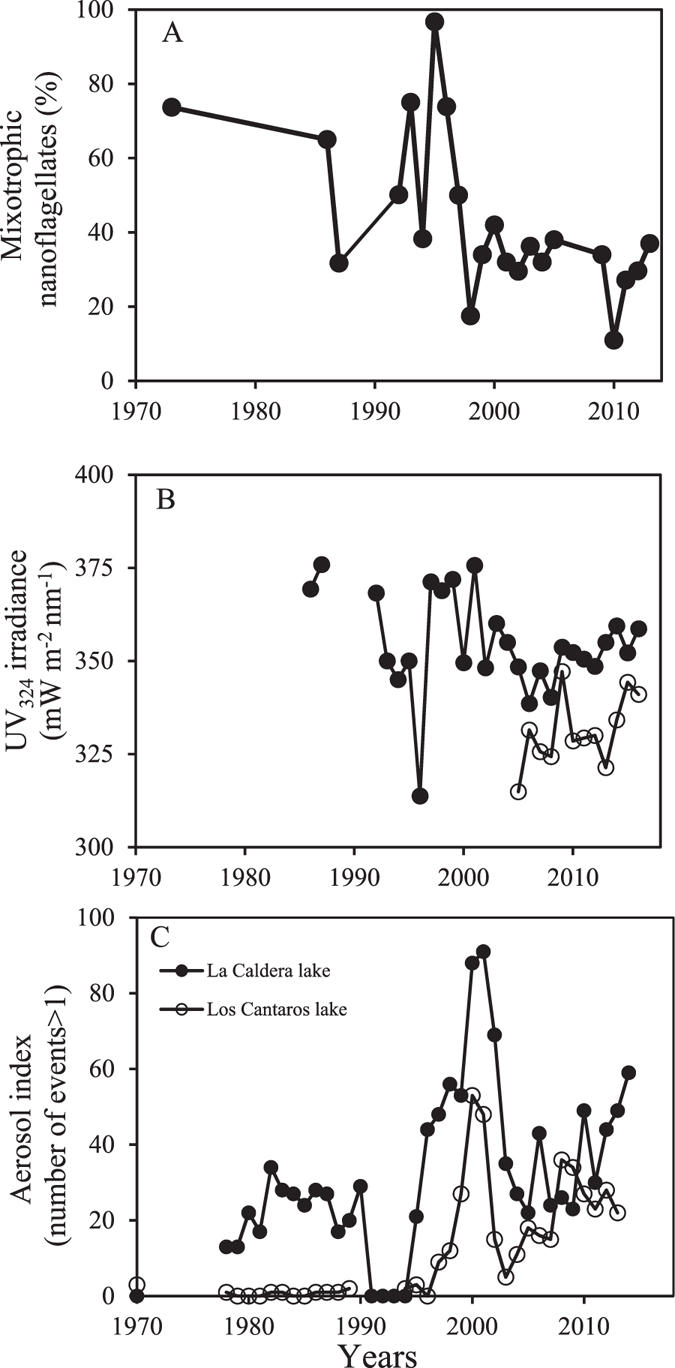
Interannual trends in mixotroph nanoflagellates (MNFs), UVR irradiance, and aerosol events. (**A**) Mean annual changes in percentage of mixotroph nanoflagellates (MNFs) during ice-free seasons between 1973 and 2014 in Lake La Caldera; (**B**) UV_324nm_ irradiance in both area lakes; (**C**) Aerosol index (AI), number events AI > 1 is sum of the daily of events AI > 1 for the ice-free period considered for each lake area and year from 1978 to 2016.

The most striking feature of the time-series for the percentage of mixotrophic nanoflagellates (MNFs) in La Caldera, UVR_324_nm irradiance, and events AI > 1 is the opposite trend found between MNFs (decreasing relative importance) and events AI > 1 (increasing) in La Caldera lake (Fig. 1). Forward stepwise regressions showed that, of all potential climatic predictors, precipitation and events AI > 1 explained a significant variance (43%) of MNFs (*p* *<* 0.001). The greater numbers of events AI > 1 were detected since late of 90’s in both areas but in Los Cántaros area were below those of the La Caldera area for most the time series (Fig. 1).

### Initial conditions of physical variables in each lake

During the experimental periods, water temperature was similar between the mesocosms and the lake for each of the study sites, and only slight temperature differences between lakes were detected (La Caldera:17 ± 1 °C, and Los Cántaros:19 ± 1 °C). At the surface, photosynthetically active radiation (PAR) was similar between the lakes, i.e. 2195 and 2120 µmol photons m^−2^ s^−1^ in La Caldera and Los Cántaros lakes, respectively. La Caldera was more transparent (kd_305_ = 0.648) than Los Cántaros (kd_305_ = 1.740). However, as the incident UV-B irradiance was higher in Los Cántaros (305 nm band: 7 µW cm^−2^ nm^−1^) than in La Caldera (305 nm band: 5 µW cm^−2^ nm^−1^), differences in underwater UV-B irradiance between container set-ups at 0.1 m below the surface were negligible.

### Effects of UVR in the mesocosm experiments

**Figure 2:**
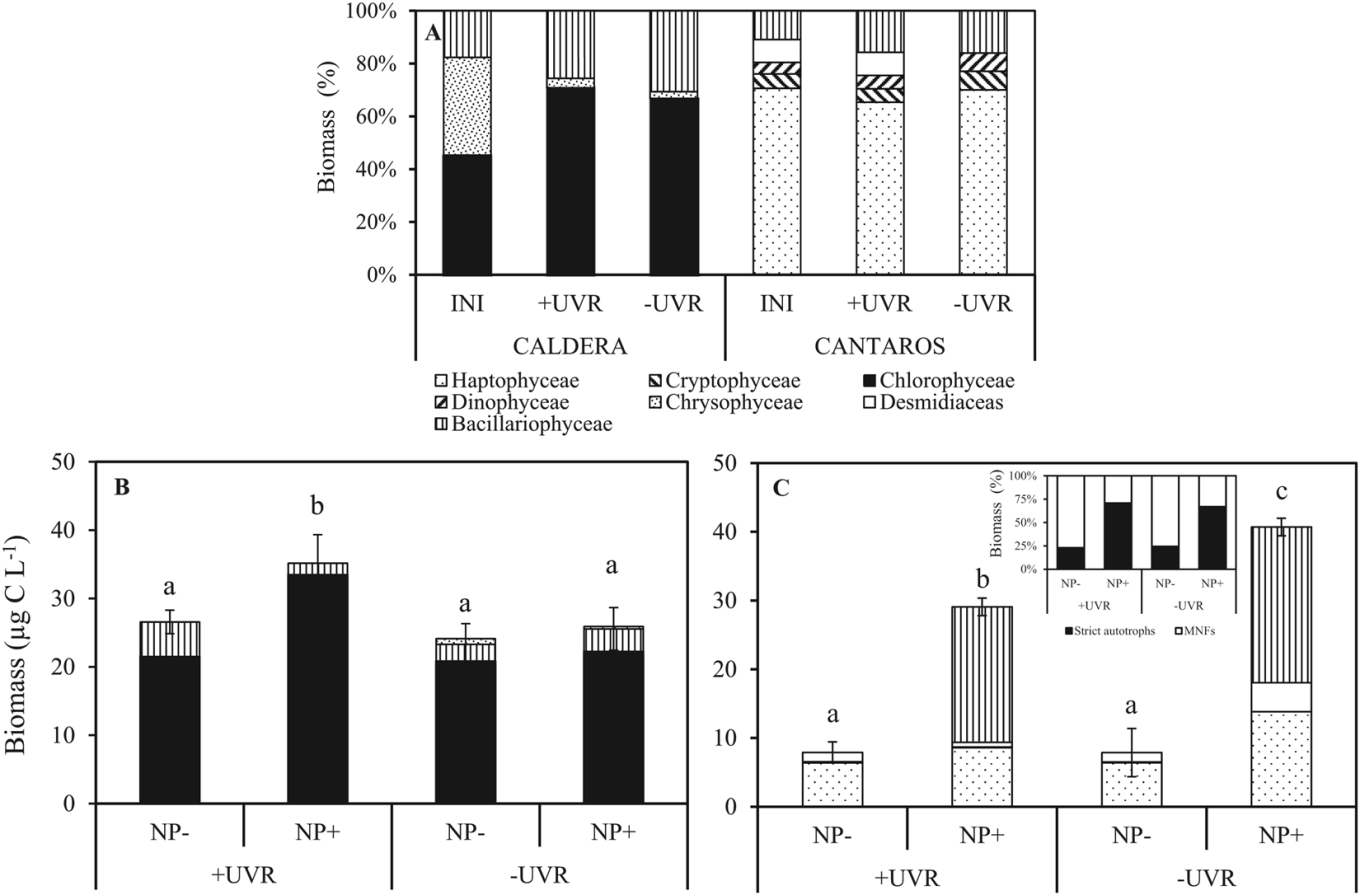
Phytoplankton taxonomical composition in mesocosms and microcosms under the experimental conditions. (**A**) Algal taxonomical composition (as biomass percentage) at initial conditions and after one month of incubation under full sunlight (+UVR) and photosynthetically active radiation (−UVR) in both lakes. Phytoplankton biomass and taxonomical composition in the experimental treatments after one week of incubation in La Caldera (**B**) and Los Cántaros (**C**). Inset figure in C represents percentage of strict autotrophs and mixotrophic nanoflagellates (MNFs) under the four treatments previously mentioned. +UVR: full sunlight; (>280 nm); −UVR: screened sunlight (>400 nm); NP−: non-nutrient added; NP+: nutrient added. Data are expressed as mean values ± SD (n = 3). Significant differences among treatments are denoted by different lower-case letters.

The phytoplanktonic community was composed of strict autotrophs (63%) mainly of Chlorophyceae (45%) and Bacillariophyceae (18%) in La Caldera and up to 78% by MNFs (Haptophyceae and Cryptophyceae) in Los Cántaros (Fig. 2A). Incubation in the mesocosms (+UVR and −UVR) for a month did not change the specific algal composition in Los Cántaros, but increased the dominance by Chlorophyceae in La Caldera regardless of the light treatment. *Monoraphidium* sp. was the dominant species in La Caldera and *Chrysochromulina parva* in Los Cántaros, reaching up to the c. 70% of biomass, respectively (Fig. 2A). The mean cell biovolume of the phytoplanktonic community was 289 µm^3^ in Los Cántaros and 92 µm^3^ in La Caldera. Other microplanktonic groups, such as ciliate or heterotrophic nanoflagellates (HNF) were not detected in La Caldera, and small mixotrophic ciliates (Oligotrichida) were present in very low abundance (<2% phytoplankton abundance) in Los Cántaros. No significant differences in Chl *a* and phytoplanktonic biomass were detected between the two light treatments. However, the sestonic C:P ratio was significantly lower under the +UVR treatment in both lakes (Table S1).

### Interactive UVR × Nutrient effects in microcosms

UVR × Nutrient interactions (sub-plot effect) significantly affected phytoplanktonic biomass in both lakes (Supplementary Table S2). While biomass was unaffected by UVR alone, the NP addition exerted a positive synergistic effect with the high values in the +UVR_NP+_ treatment in La Caldera but in the −UVR_NP+_ treatment in Cántaros (i.e. a positive antagonistic effect; Supplementary Table S3).

**Figure 3:**
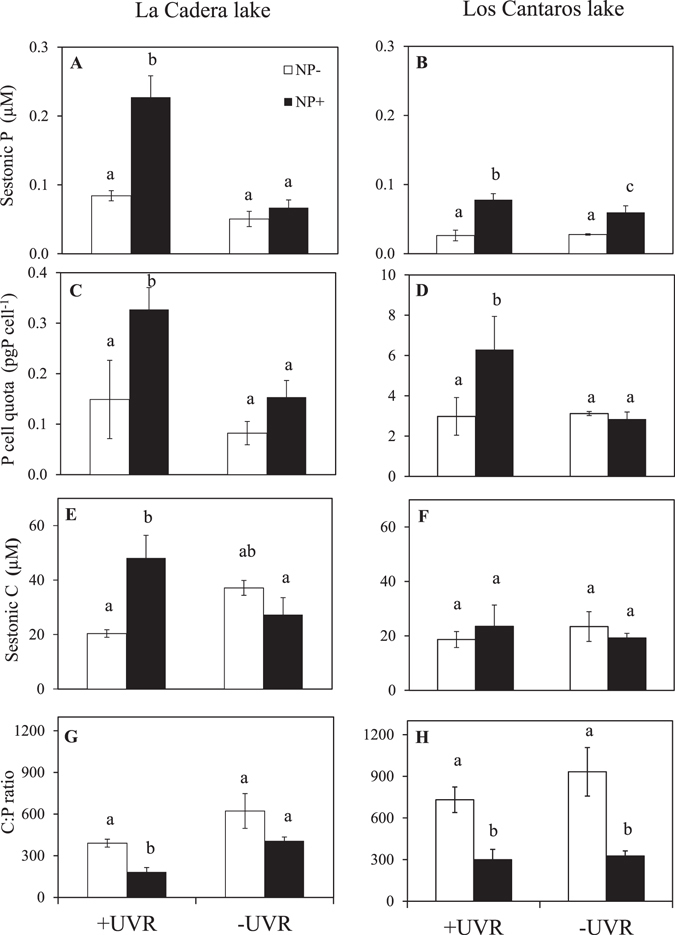
Elemental content of the sestonic fraction under the experimental conditions. Stoichiometric variables of sestonic fraction under radiation [full sunlight (+UVR) and photosynthetically active radiation (−UVR)] and nutrient treatments [nutrient-enriched (NP_+_) and non-enriched (NP_−_)] in La Caldera and Los Cántaros lakes. Sestonic Phosphorous (P) (**A**,**B**); P cell quota (**C**,**D**); sestonic Carbon (**C**) (**E**,**F**); sestonic C:P ratio (**F**,**G**). Data are expressed as mean values ± SD (n = 3). Significant differences among treatments are denoted by different lower-case letters.

For the sestonic P and P cell quota, UVR × Nutrient interaction (sub-plot effect) exerted a significant effect in both lakes (Supplementary Table S2), with the values of these variables being highest under +UVR_NP+_ conditions (Fig. 3). While UVR, under unenriched conditions, did not affect the sestonic P and P cell quota in any lake (Bonferroni test, Fig. 3A,B), NP addition significantly increased the sestonic P regardless of the light treatment in Los Cántaros. This did not occur in La Caldera, where the sestonic P increased only under UVR (Fig. 3A,B). The UVR × Nutrient effect on sestonic C proved significant only in La Caldera (Supplementary Table S2), with the highest values in +UVR_NP+_ treatments (Fig. 3E,F). Therefore, a different response of the C:P ratio to both factors was found in each lake. While joint UVR and Nutrient lowered the C:P ratio in La Caldera, Nutrient was the only factor that reduced the C:P ratio in Los Cántaros (Supplementary Table S2; Fig. 3G,H). It is noticeable that under unenriched conditions, the C:P ratio was lower in La Caldera than in Los Cántaros, indicating a stronger sestonic P limitation in Los Cántaros. In fact, sestonic P was two-fold higher in La Caldera than in Los Cántaros for most treatments, although the P cell quota was 20-fold higher in Los Cántaros than in La Caldera, regardless of the nutrient treatment.

As for sestonic P, a significant UVR × Nutrient effect (sub-plot effect) was found on phytoplanktonic biomass, Chl *a*, and Chl *a*: C ratio in both lakes (Fig. 2; Table S1 and Supplementary Fig. S2). The interactive effects were positive synergistic (values more positive than predicted additively; Supplementary Table S3) in La Caldera, with the highest values of Chl *a*, Chl *a*: C ratio under +UVR_NP+_ conditions (Supplementary Fig. S2). By contrast, the interactive effects were positive antagonistic in Los Cántaros (values of each variable less than predicted additively; Supplementary Table S3).

**Figure 4:**
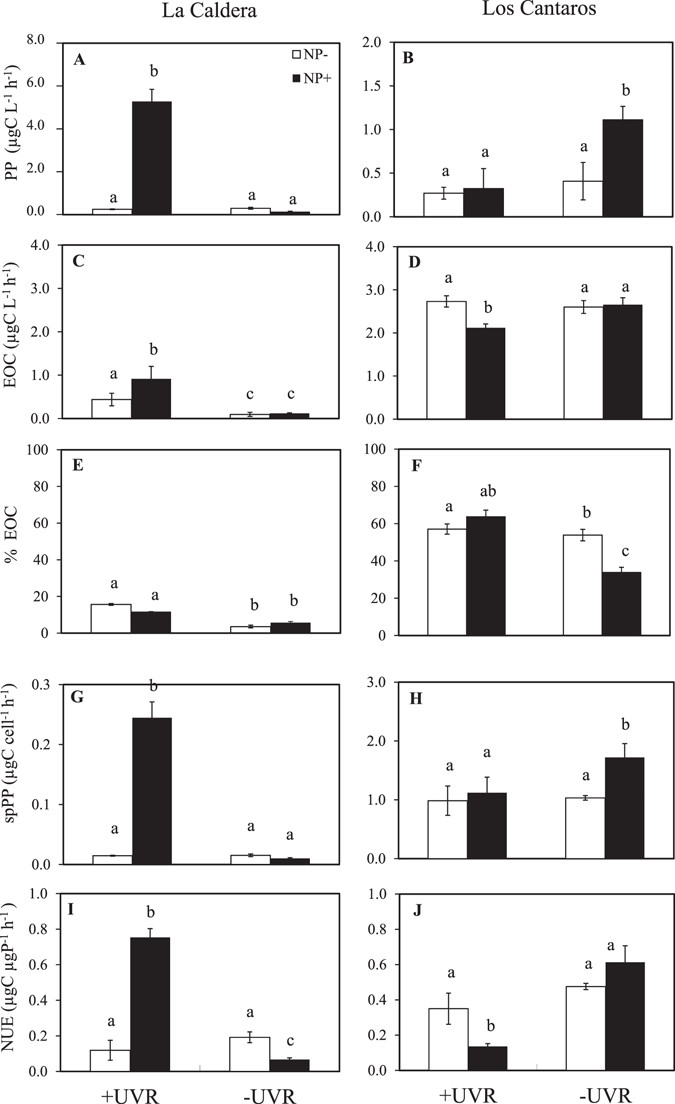
Metabolic variables of the phytoplankton community under the experimental conditions. Primary production, excreted organic carbon (%) and productivity under radiation [full sunlight (+UVR) and photosynthetically active radiation (−UVR)] and nutrient treatments [nutrient-enriched (NP+) and non-enriched (NP−)] in La Caldera and Los Cántaros lakes. Primary production (PP) (**A**,**B**); excretion of organic carbon rates (EOC) (**C**,**D**); percentage of excretion of organic carbon (%EOC) (**E**,**F**); specific cell productivity (spPP) (**G**,**H**); photosynthetic nutrient-use efficiency (PNUE) (**I**,**J**). Data are expressed as mean values ± SD (n = 3). Significant differences among treatments are denoted by different lower-case letters.

With regard to functional variables, UVR × Nutrient also exerted a significant effect on PP in both lakes (Table 3S). The interactive effect was positive synergistic in La Caldera and positive antagonistic in Los Cántaros (Fig. 4; Supplementary Table S3). Under unenriched conditions, UVR did not affect PP, but NP-addition significantly stimulated PP under UVR in La Caldera, and in the absence of UVR in Los Cántaros (Bonferroni test, Fig. 4A,B). The UVR × Nutrient effect on the EOC and %EOC was significant in Los Cántaros but not in La Caldera (Supplementary Table S4). Under unenriched conditions, UVR augmented the EOC and %EOC in La Caldera (Bonferroni test, Fig. 4C–F), whereas the NP addition diminished the %EOC in the absence of UVR in Los Cántaros (Fig. 4E,F). EOC and %EOC were higher in Los Cántaros than in La Caldera for all treatments. A significant negative relationship between the %EOC and C:P ratio was found only in La Caldera (R^2^ = 0.46, *p* = 0.015).

The specific productivity (spPP) was one order of magnitude higher in Los Cántaros than in La Caldera (Fig. 4G,H) because of algal abundance was more than one order of magnitude higher in La Caldera than Los Cántaros (data not shown) whereas PP values were within a similar range in both lakes. As for PP, under unenriched conditions, UVR did not exert a significant effect on spPP in any lake, but UVR × Nutrient (subplot effect) was significant only in La Caldera, with the highest spPP in the +UVR_NP+_ treatment (Bonferroni test, Supplementary Table S4; Fig. 4G).

Finally, although the UVR × Nutrient effect on P cell quota, and sestonic P were positively synergistic in both lakes, the interactive effect on PNUE was positively synergistic in La Caldera but negatively synergistic in Los Cántaros (Fig. 4I,J; Supplementary Tables S2, S3). As a result, the P cell quota negatively correlated to PNUE in Los Cántaros (R^2^ = 0.66, *p* = 0.001), but not in La Caldera.

**Figure 5:**
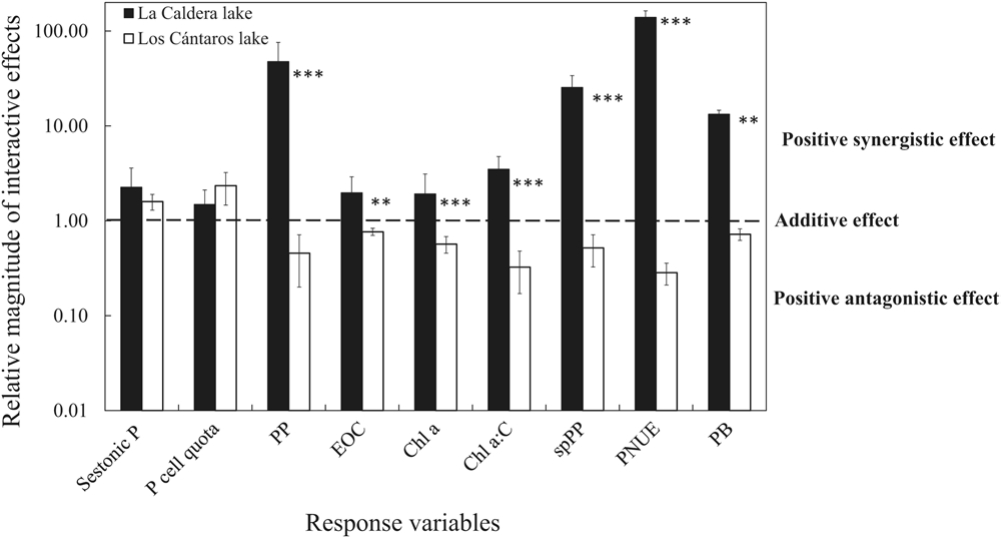
Nature and magnitude of the observed interactive impacts of UVR, and nutrient enrichment. Sestonic P, P cell quota, primary production (PP), excretion of organic carbon (EOC), Chlorophyll a (Chl a), specific cell productivity (spPP), photosynthetic nutrient-use efficiency (PNUE), and phytoplanktonic biomass (PB) (more details in Table S3).

In summary, our results partially agree with our initial hypothesis that nutrient inputs unmasked the negative UVR effect (with a positive antagonistic or negative synergistic effect), which was supported only in Los Cántaros (Fig. 5). Thus, our results reveal a consistently greater UVR sensitivity of functional and structural variables after nutrient addition in Los Cántaros than in La Caldera.

## Discussion

The main goal of this study was to anticipate how an increase in mineral nutrients related to the dust-aerosol transport to highly UVR-exposed ecosystems may affect the stoichiometry, metabolism, and structure of primary producers in lakes with contrasting exposure to dustaerosol deposition. For this purpose, we selected two lakes that represent different scenarios in the context of global change: one in the Sierra Nevada, Spain, with high UVR exposure and where nutrient inputs from nearby desert areas (mainly the Sahara Desert) are frequent^11, 38^, and the other in the northern Patagonian Andes, where high UVR exposure but minor allochthonous atmospheric nutrient loads drive the planktonic community development^39^. This approach is in line with the current demand of studies incorporating the temporal extent and action scales of press (UVR disturbance) and pulse (nutrient disturbance) into multiple stressors research, as a realistic way to evaluate the complex effect of global change^40^. In this way, our findings show higher UVR vulnerability of primary producers after a nutrient pulse in Los Cántaros lake than in La Caldera lake. In fact, the UVR effects after a nutrient pulse proved inhibitory for almost all response variables (except for sestonic P and P cell quota) in Los Cantaros lake but were stimulatory in La Caldera lake. Therefore, the difference in the temporal action scale of resource pulses between the two lakes might have triggered physiological mechanisms of acclimation or adaptive responses of phytoplankton of La Caldera lake that undergoes more intense and frequent pulses not operating in Los Cántaros lake. Likewise, our findings lead us to anticipate the loss of the dominance of MNFs in a potential scenario of increased transport of mineral nutrients to oligotrophic areas in the Southern Hemisphere (see ref. 25). This prediction is based on (i) the proportional decrease of MNFs after a nutrient pulse (Fig. 2 inset) along with the decrease of the bacterivory rates after nutrient pulse (Supplementary Fig. S3); both findings are in agreement with the notion that MNFs are better adapted to P-limited and stressful light conditions than are strict autotrophs^12, 41^; (ii) the loss of MNFs after P pulses reported in previous studies through an experimental nutrient-addition gradient^12, 42^ or along a natural gradient of increasing trophic state^43^; and (iii) the inverse relationship between MNFs and the intensity of aerosol events registered over a long-term scale (1973–2014 years) in lake La Caldera (Fig. 1).

In agreement with our initial hypothesis, nutrient pulses unmasked the UVR damage in the Los Cantaros lake, as the greatest values of most of the response variables analysed occurred in the absence of UVR after the nutrient pulse (positive antagonistic effect or even a negative synergistic effect, Supplementary Table S3, Fig. 5). This interactive effect has been a consistent response to UVR and nutrients when phytoplanktonic communities are flagellate dominated in Boreal lakes^34, 44^, Mediterranean lakes^24, 36^, and coastal ecosystems^37^. In turn, contrary to our initial hypothesis, UVR and nutrients jointly stimulated all the response variables (positive synergistic effect) including stoichiometric (e.g. sestonic P, P cell quota), functional (PP, PNUE), and structural (Chl *a* and, with less magnitude, phytoplankton biomass) variables, in lake La Caldera.

The specific reasons why MNFs in Los Cántaros were more sensitive to UVR after nutrient inputs might include the following: i) The less efficient CO_2_-concentrating mechanisms (CCMs) of Prymnesiophyceae (Haptophyceae) and consequently the high dependence on the diffusive CO_2_ entry for photophynthesis^45, 46^. Supporting this interpretation, we found a negative relationship between PNUE and P cell quota and a decrease of PNUE after nutrient addition under UVR (i.e. a negative synergistic effect, Fig. 4J, Supplementary Table S3), this being consistent with the decrease in inorganic C incorporation by phytoplankton found here; ii) the higher energy demands of mixotrophs necessary to maintain their concomitant phagocytic and photosynthetic metabolisms^47, 48^ could constrain their growth compared to other algal groups; iii) there was a lack of a metabolic protective strategy via C release such as that developed by Chlorophyceae (strict autotrophs) in La Caldera (see below). This is supported by the low EOC values under UVR and nutrients (negative synergistic effect) and the absence of a relationship between sestonic C:P ratio and %EOC in Los Cántaros.

By contrast, in La Caldera, the dominance of strict autotrophs (Chlorophyceae, *Monoraphidium* sp.) under UVR and nutrient addition suggests an evolutionary adaptation of this kind of phytoplanktonic community to conditions of high UVR irradiance and intense nutrient pulses linked to dust aerosol from the Sahara Desert, decreasing MNFs, as is observed over a long-term scale of the observational data (Fig. 1) and in previous experimental approaches^42^. This interpretation is also supported by: i) the increase in PP, which is consistent with a potential UVA stimulation of photosynthesis, as shown by Gao *et al*.^49^ and with maximum HCO_3_^−^ utilization in the blue and UVA regions of the light spectrum by *Monoraphidium braunii* ^50^; ii) the negative relationship between the C:P ratio and the %EOC, implying that algae with a more balanced C:P ratio after a nutrient pulse released part of the organic C produced as a protective mechanism to prevent the photosystem damage under high light irradiance^24, 51^; and iii) the highest EOC values coinciding with a higher productivity (spPP) in agreement with the hypothesis that C release provides a mechanism to maintain the potential photosynthetic capacity^24, 36, 52^. Therefore, we propose that these mechanisms enable these strict autotrophic algae to grow in response to the steady increase in the number of intense nutrient-pulse events since the late 1990s^11^ (see Fig. 1), whereas frequent low-intensity events favour the persistence of MNFs^38^. On the other hand, the greater EOC under UVR and nutrient inputs might explain the mismatch between the strong stimulatory effect on PP and the minor stimulatory effect on biomass found (see ref. 53).

## Conclusions

Our results show higher UVR vulnerability after nutrient addition of primary producers in the southern Patagonian lake (Los Cántaros), where the atmospheric nutrient inputs are lower compared to those in lake La Caldera (Sierra Nevada, Spain). The potential loss of nanoflagellates with a high proportion of mixotrophic species (e.g. *Chrysochromulina* sp.) would affect ecosystem functioning because the mixotrophs constitute a by-pass in the C flux between the microbial loop and the grazing chain in freshwater^54^ and marine ecosystems^55, 56^. At regional scale, future increases in the intensity of aerosol events (or frequency of intense events) from desert and desertified areas would result in the biological impoverishment associated with the loss of these key functional species (MNFs), altering the C flux from microbial to the grazing chain in clear-water ecosystems.

## Methods

### Model ecosystems

La Caldera is a high-mountain lake located above the tree line (3050 m a.s.l.) in Sierra Nevada National Park (southern Spain, 36°55′–37°15′N, 2°31′–3°40′W). Los Cántaros, a mountain lake located at 1000 m.a.s.l in Andes mountain range (north-eastern Patagonia, Argentina, 41°00′S, 71°49′W), is of glacial origin. Both lakes are oligotrophic (Chl a is <1 µg L^−1^), highly transparent (>10% of photosynthetically active radiation [PAR, 400–700 nm] penetrate to maximum depth) and DOC <1 mg C L^−1^ ^31, 57^. La Caldera undergoes inputs of nutrient-rich Saharan dust^22, 58^ whereas in Los Cántaros the atmospheric P loads are negligible^22^ (more details in Supplementary text S1).

### Long-term study of phytoplankton in La Caldera

Phytoplankton were collected over different intervals (from 4-d to monthly intervals) during the ice-free period in Lake La Caldera during 1973–2014 years. Although the sampling program had some disruptions, in each decade from 2 to 6 years were sampled. Samples were gathered with a Van Dorn sampler at the deepest point of the lake from four depths (subsurface, two middle depths, and 0.5 m above bottom) and preserved using Lugol’s reagent (approx. 1% vol/vol). All taxa were identified to the species level under an inverted microscope. Analysis protocols and original data are fully reported elsewhere^32, 56, 59^.

### Climatic data

The AI value > 0.5 was considered to represent a deposition event as a proxy for dry dust deposition and UV irradiance environment over each lake area, we used the area-averaged UV aerosol index (AI) and UV irradiance 324 nm (local noon) data downloaded from the Giovanni database (v 4. 21; http://giovanni.gsfc.nasa.gov/giovanni) developed by the Goddard Earth Sciences Data and Information Services Centre of the National Aeronautics and Space Administration (NASA) for Sierra Nevada range (36°55′–37°15′N; 2°31′–3°40′W) and an equivalent area surrounding Los Cántaros lake. Available daily data (provided by TOMS Nimbus-7, TOMS EarthProbe and OMI satellites) were collected for the free-ice season for each lake (Caldera: 1 June to 15 November; Cántaros, 1 December to 15 May) spanning from 1978 to 2016 (see Supplementary text S2). Total precipitation (rainfall and snowfall) was determined from annual averages of weekly collected samples at a nearby meteorological station in Trevélez (Sierra Nevada) continuously measured by the Agencia Estatal de Meteorología (AEMET; original data at http://www.aemet.es).

### Experimental design

he study was carried out from July to August 2007 in Lake La Caldera (Northern Hemisphere) and from January to February 2008 in Lake Los Cántaros (Southern Hemisphere). In both lakes, the experiment was performed in the middle of summer to attempt incubation days with clear skies and stable weather. To evaluate a single UVR effect on the microplanktonic community, each treatment consisted of mesocosms made of clear polyethylene tubes (0.7 m in diameter × 5 m in length), closed at the bottom, with total volume of 2 m^3^. The mesocosms were filled by pumping screened (45-µm mesh, to avoid the indirect effect on microplankton driven by herbivorous consumers) lake water within the 3-m depth (within photic layer affected by >5% UVB) and they were incubated in each lake for one month. Three of the mesocosms received the full spectrum of solar radiation (+UVR treatment) and the other three received only photosynthetically active radiation (PAR; −UVR treatment; more details on the optical properties of mesocosms in Supplementary text S3).

After one month, water from each of the six mesocosms, vertically homogenized by using a round plastic disc fixed to a rope, was used to fill small closed microcosms of 20 L (0.2 m in length, 0.4 m in diameter) constructed from the same polyethylene as that of the mesocosms (12 microcosms, i.e. 6 UVR-transparent and 6 screened). Three microcosms of each light treatment received phosphorus (Na_2_HPO_4_) and nitrogen (NH_4_NO_3_) to around double the total P concentration in the corresponding lake, maintaining a molar N:P ratio of ~30. These nutrient additions were meant to mimic the mean TN:TP ratios detected after natural atmospheric deposition events in La Caldera, and were maintained to simulate similar nutrient loads in Los Cántaros. The other three containers of each light treatment (3 UVR-transparent and 3 screened) received no nutrient addition.

The experiment is a split-plot design, with light treatments applied at the plot level and nutrient treatments at the subplot level with three replicates per treatment: −UVR_NP−_: screened sunlight (>400 nm); −UVR_NP+_: screened sunlight (>400 nm) + nutrient addition; +UVR_NP−_: full sunlight; +UVR_NP+_: full sunlight + nutrient addition (Supplementary Fig. S1). The 12 containers were then incubated for one week at ca. 0.1 m below the surface, corresponding to 75% of surface solar radiation (light inside the containers).

### Physical analyses

Underwater irradiance and temperature was measured with BIC compact 4-channel in La Caldera and PUV-500B submersible radiometers in Los Cántaros (Biospherical Instruments, CA, USA). Vertical profiles of diffuse attenuation coefficients for downward irradiance (kd) were determined from the slope of the linear regression of natural logarithm of downwelling irradiance *vs*. depth for each wavelength range considered.

### Sampling and laboratory analysis

The six large mesocosms and the 12 microcosms were sampled (1 L) for sestonic C, N, and P and for the microplankton abundance, Chl *a* concentration, and PP. Samples for C, N, P, and Chl *a*, were immediately transported to the laboratory in cold, dark, and thermally insulated containers. In the laboratory, a volume of 300 mL from each sample was filtered onto pre-combusted GF/F Whatman filters (450 °C for 1.5 h) for seston elemental analysis, filters were dried at 60 °C for 48 h and stored at −20 °C until analysed. C and N were analysed on a Perkin Elmer 2400 (Perkin Elmer, USA) (La Caldera) and Thermo Finnigan EA1112 (Thermo Finnigan, Italy) CHN (Los Cántaros) elemental analyzers and P was analysed with persulfate digestion followed by molybdate reaction^60^. Chl a was measured by filtering a volume of 300 mL at <100 mm Hg onto Whatman GF/F glass microfibre filters (25 mm in diameter) and extracting in a 90% acetone solution (at 4 °C for 24 h in the dark). Determinations were made in a Turner AU 10 fluorometer in Los Cántaros and LS 55 Perkin Elmer fluorometer in La Caldera^60^. To quantify abundance of phytoplankton, heterotrophic nanoflagellates (HNF), and small ciliates, we followed the procedures described in Medina-Sánchez *et al*.^12^ (Supplementary text S4).

### Primary-production procedure

PP was measured by the ^14^C method proposed by Steeman-Nielsen^61^. Sets of four 50-ml quartz flasks (three clear and one dark), with 0.37 MBq of NaH^14^CO_3_ (specific activity: 310.8 MBq mmol^−1^, DHI Water and Environment, Germany) added to each flask, were incubated *in situ* at 75% of 305 nm wavelength (UVB) surface incident irradiance for 4 h symmetrically distributed around noon. All flask sets were horizontally held during the incubations. Total primary production was measured as total organic carbon (TOC) by acidifying a 4-ml subsample. Particulate PP was determined by filtering an aliquot of 45 mL through 1.0-µm pore-size Nuclepore filters of 25-mm diameter. To minimize cell breakage, we applied low pressure (<100 mm of Hg). The filters were placed in scintillation vials and the DI^14^C was removed by adding 100 µl of 1 N HCl. The filtrate (<1 µm, excretion of organic carbon (EOC)) was also collected and treated as described above for the TOC (more details in Carrillo *et al*.^51^). The percentage of excretion of organic carbon (%EOC) by algae was estimated as:

1 $$ \% {\rm{EOC}}=100{\rm{x}}\,({\rm{EOC}}/{\rm{TOC}})$$

The specific cell productivity (spPP) was calculated as POC normalized by cell abundance. The P cell quota was the sestonic P content divided by cell abundance. The photosynthetic nutrient-use efficiency (PNUE, *sensu* ^62^) was estimated as a quotient between POC and sestonic P content.

### Data calculation and statistical analyses

The direction and magnitude of the interactive effect for each response variable in both ecosystems was calculated by comparing the values in the combined UVR × Nutrient treatment (non-additive effect) with their expected additive value based on the sum of individual effects terms [e.g. −UVR_NP−_ + (+UVR_NP_ − (−UVR_NP−_)) + (−UVR_NP+_ − (−UVR_NP−_))]. The nature of interactive effect was defined following Piggott *et al*.^13^ (Fig. 2 therein), a positive synergistic interaction was defined as the case in which the value of the combined treatment (non-additive effect) is higher than the expected additive value and greater than absolute value resulting from each individual effect. By contrast, positive antagonistic interaction was defined as the case in which the value of the combined treatment (non-additive effect) is lesser than the expected additive value but higher (or equal) than the absolute value resulting of the strongest individual effect. The negative synergistic interaction, as the case in which the value of the combined treatment (non-additive effect) was lower than the expected additive value and lower than the absolute value resulting from the strongest individual effect. The magnitude and direction of the response of each variable to the interactive effect was calculated as the quotient between the observed (+UVR_NP+_ values) and expected additive effects. We used propagation errors to calculate the relative magnitude of the interactive effect.

Forward stepwise regression analyses were conducted to identify the climatic drivers (water temperature, annual precipitation, UVR irradiances, and high intensity of AI; number of events >1) controlling the long-term dynamics of MNFs (as % of total phytoplankton abundance) in La Caldera. Linearity and orthogonality among independent variables were verified by previous correlation analysis and controlled by specifying 0.6 as the minimum acceptable tolerance (StatSoft Inc, 2005)^63^.

The UVR effect on sestonic C, N, and P and their ratios, Chl *a*, and phytoplankton biomass were analysed with a t-test for the mesocosm experimental data. For the microcosm experiments, the effects of UVR as the main-plot effect, and UVR × Nutrient interaction as the sub-plot effect on structural (biomass, Chl *a*) stoichiometric (C, N, P, C:P, and C:N) and functional (PP, EOC rate, and %EOC) variables were assessed by two-way split-plot of repeated measures analysis of variance (RM-ANOVA)^64^ after verifying the assumptions required by the RM-ANOVA. When the interactive effect of the two factors on the response variable was significant, the *post hoc* Bonferroni test was used to determine the effect of each factor. To explore the relationship between stoichiometry and functional variables, we conducted a linear regression of sestonic C:P vs. %EOC, and PNUE vs. P cell quota for the experimental data in each lake. The statistical analyses were performed using Statistica 7.1 for Windows software (StatSoft Inc).

## Electronic supplementary material

Supplementary Information

## References

[1] Crain CM, Kroeker K, Halpern BS (2008). Interactive and cumulative effects of multiple human stressors in marine systems. Ecol. Lett..

[2] Darling S, Cote IM (2008). Quantifying the evidence for ecological synergies. Ecol. Lett..

[3] Boyd PW, Hutchins DA (2012). Understanding the responses of ocean biota to a complex matrix of cumulative anthropogenic change. Mar. Ecol. Progr. Ser..

[4] Gunderson AR, Armstrong EJ, Stillman. JH (2016). Changing World: The Need for an Improved Perspective on Physiological Responses to the Dynamic Marine Environment. Annu. Rev. Mar. Sci..

[5] Gaol K (2012). Rising CO_2_ and increased light exposure synergistically reduce marine primary productivity. Nat. Clim. Chang..

[6] Boyd, P. W., Lennartz, S. T., Glover D. M. & Doney, S. C. Biological ramifications of climate-change mediated oceanic multi-stressors. *Nat. Clim. Chang*. doi:10.1038/Nclimate 2441 (2014).

[7] Kneitel JM, Chase JM (2004). Trade-offs in community ecology: linking spatial scales and species coexistence. Ecol. Lett..

[8] Blanck, H. *Human Ecology and Risk Assessment*. **8**, 1003–1034 (2002).

[9] Vinebrooke RD (2003). Trophic dependence of ecosystem resistance and species compensation in experimentally acidified lake 302S (Canada). Ecosystems.

[10] Petchey OL (2004). Species loss and the structure and functioning of multitrophic aquatic systems. Oikos.

[11] Bullejos F, Carrillo P, Villar-Argaiz M, Medina-Sánchez JM (2010). Roles of phosphorus and ultraviolet radiation in the strength of phytoplankton-zooplankton coupling in a Mediterranean high-mountain lake. Limnol. Oceanogr..

[12] Medina-Sánchez JM (2013). Maximum in the middle: Nonlinear response of microbial plankton to ultraviolet radiation and phosphorus. PLoS One.

[13] Piggott JJ, Townsend CR, Matthae CD (2015). Reconceptualizing synergism and antagonism among multiple stressors. Ecol. Evol..

[14] Jackson M. C., Loewen, C. J. G., Vinebrooke, R. D. & Chimimba, C. T. Net effects of multiple stressors in freshwater ecosystems: a meta-analysis. *Glob. Chang. Biol*. doi:10.1111/gcb.13028 (2015). 10.1111/gcb.13028 26149723

[15] Salbu B, Rosseland BO, Oughton DH (2005). Multiple stressors - A challenge for the future. J. Environ. Monit..

[16] Neale, P. J., Helbling, E. W. & Zagarese, H. E. Modulation of UVR exposure and effects by vertical mixing and advection, in: *UV Effects in Aquatic Organisms and Ecosystems*. (eds Helbling, E. W. & Zagarese, H. E.) 108–134 (Royal Society of Chemistry, 2003).

[17] Harrison JW, Smith REH (2009). Effect of ultraviolet radiation on the productivity and composition of freshwater phytoplankton communities. Photochem. Photobiol Sci..

[18] Häder, D. P. *et al*. Effects of UV radiation on aquatic ecosystems and interactions with other environmental factors. *Photochem. Photobiol. Sci*. doi:10.1039/c4pp90035a (2014). 10.1039/c4pp90035a 25388554

[19] Beardall J, Slobodanka S, Gao K (2014). Interactive effects of nutrient supply and other environmental factors on the sensitivity of marine primary producers to ultraviolet radiation: implications for the impacts of global change. Aquat. Biol..

[20] Neff JC (2008). Increasing eolian dust deposition in the western United States linked to human activity. Nat. Geosci..

[21] Mahowald N (2011). Aerosol indirect effects on biogeochemistry and climate. Science.

[22] Mladenov, N. *et al*. Dust inputs and bacteria influence dissolved organic matter in clear alpine lakes. *Nat. Commun*. **2**, doi:10.1038/ncomms1411 (2011). 10.1038/ncomms1411 PMC3144587 21792184

[23] Mahowald N (2008). The global distribution of atmospheric phosphorus deposition and anthropogenic impacts. Glob. Biogeochem.Cycles.

[24] Carrillo P, Delgado-Molina JA, Medina-Sánchez JM, Bullejos FJ, Villar-Argaiz M (2008). Phosphorus inputs unmask negative effects of ultraviolet radiation on algae in a high mountain lake. Glob. Chang. Biol..

[25] Brahney, J., Mahowald, N., Ward, D. S., Ballantyne, A. P. & Neff, J. C. Is atmospheric phosphorus pollution altering global alpine Lake stoichiometry? *Glob. Biogeochem. Cycles* **29**, doi:10.1002/2015GB005137 (2015).

[26] Williamson CE (2014). Solar ultraviolet radiation in a changing climate. Nat. Clim. Chang..

[27] Herman, J. R. Global increase in UV irradiance during the past 30 years (1979–2008) estimated from satellite data. *J. Geophys. Res*. **115**, doi:10.1029/2009JD012219 (2010).

[28] World Meteorological Organization (WMO/UNEP). *Scientific Assessment of Ozone Depletion*: 2010. Executive Summary. Prepared by the Scientific Assessment Panel of the Montreal Protocol Substances that Deplete the Ozone Layer, United Nations Environmental Protection Agency (2010).

[29] Aubriot L, Conde S, Sommaruga R (2004). Phosphate uptake behaviour of natural phytoplankton during exposure to solar ultraviolet radiation in shallow coastal lagoon. Marine Biol..

[30] Hessen DO, Frigstad H, Færøvig PJ, Wojewodzic MW, Leu E (2011). UV radiation and its effects on P-uptake in arctic diatoms. J. Exp. Mar. Biol. Ecol..

[31] Carrillo P (2015). Synergistic effects of UVR and simulated stratification on commensalistic algal-bacterial relationship in two optically contrasting oligotrophic Mediterranean lakes. Biogeosciences.

[32] Durán C, Medina-Sánchez JM, Herrera G, Carrillo P (2016). Changes in the phytoplankton-bacteria coupling triggered by joint action of UVR, nutrients, and warming in Mediterranean high-mountain lakes. Limnol. Oceanogr..

[33] Medina-Sánchez JM, Villar-Argaiz M, Carrillo P (2006). Solar radiation-nutrient interaction enhances the algae-bacteria link in a high-mountain lake. Limnol. Oceanogr..

[34] Xenopoulos MA, Frost C, Elser JJ (2002). Joint effects of UV radiation and phosphorus supply on algal growth rate and elemental composition. Ecology.

[35] Doyle SA, Saros JE, Williamson C (2005). Interactive effects of temperature and nutrient limitation on the response of alpine phytoplankton growth to ultraviolet radiation. Limnol. Oceanogr..

[36] Korbee N (2012). Effects of ultraviolet radiation and nutrients on the structure-function of phytoplankton in a high mountain lake. Photochem. Photobiol. Sci..

[37] Carrillo P (2015). Interactive effect of UVR and phosphorus on the coastal phytoplankton community of the Western Mediterranean Sea: Unravelling eco-physiological mechanisms. Plos One.

[38] Cabrerizo MJ, Medina-Sánchez JM, González-Olalla JM, Villar-Argaiz M, Carrillo P (2016). Saharan dust inputs and high UVR levels jointly alter the metabolic balance of marine oligotrophic ecosystems. Sci Rep..

[39] Modenutti BE (2013). Effect of volcanic eruption on nutrients, light, and phytoplankton in oligotrophic lakes. Limnol. Oceanogr..

[40] Cheng BS (2015). Testing local and global stressor impacts on a coastal foundation species using an ecologically realistic framework. Glob. Chang. Biol..

[41] Modenutti BE (2014). Mixotrophy in Argentina freshwaters. Advanc. Limnol..

[42] Delgado-Molina JA, Carrillo P, Medina-Sánchez JM, Villar-Argaiz M, Bullejos FJ (2009). Interactive effects of phosphorus loads and ambient ultraviolet radiation on the algal community in a high-mountain lake. J. Plankton Res..

[43] Saad JF, Unrein F, Tribelli PM, López N, Izaguirre I (2016). I. Influence of lake trophic conditions on the dominant mixotrophic algal assemblages. J. Plankton Res..

[44] Xenopoulos MA, Frost PC (2003). UV radiation, phosphorus, and their combined effects on the taxonomic composition of phytoplankton in a boreal lake. J. Phycol..

[45] Raven JA, Giordano M, Beardall J, Maberly SC (2011). Algal and aquatic plant carbon concentrating mechanisms in relation to environmental change. Photosynth. Res..

[46] Raven JA, Giordano M, Beardall J, Maberly SC (2012). Algal evolution in relation to atmospheric CO_2_: carboxylases, carbon-concentrating mechanisms and carbon oxidation cycles. Philos. Trans. R. Soc. B.

[47] Raven JA (1997). Phagotrophy in phototrophs. Limnol. Oceanogr..

[48] Wilken S, Schuurmans MJ, Matthijs HCP (2014). Do mixotrophs grow as photoheterotrophs? Photophysiological acclimation of the chrysophyte Ochromonas danica after feeding. New Phytol..

[49] Gao K (2007). Solar UV radiation drives CO_2_ fixation in marine phytoplankton: a double-edged sword. Plant Physiol..

[50] Giráldez N, Aparicio PJ, Quiñones MA (2000). Limiting CO_2_ levels induce a blue light‐dependent HCO_3_^−^ uptake system in *Monoraphidium braunii*. J. Exp. Bot..

[51] Carrillo P, Medina-Sánchez JM, Villar-Argaiz M (2002). The interaction of phytoplankton and bacteria in a high-mountain lake: importance of the spectral composition of solar radiation. Limnol. Oceanogr..

[52] Berman-Frank I, Dubinsky Z (1999). Balanced growth in aquatic plants: Myth or reality?. Bioscience.

[53] Finkel ZV (2010). Phytoplankton in a changing world: cell size and elemental stoichiometry. J. Plankton. Res..

[54] Medina-Sánchez JM, Villar-Argaiz M, Carrillo P (2004). Neither with nor without you: a complex algal control on bacterioplankton in a high-mountain lake. Limnol. Oceanogr..

[55] Mitra A (2014). The role of mixotrophic protists in the biological carbon pump. Biogeosciences.

[56] Ptacnik R (2016). A light-induced shortcut in the planktonic microbial loop. Sci. Rep..

[57] Morris D (1995). The attenuation of solar UV radiation in lakes and the role of dissolved organic carbon. Limnol. Oceanogr..

[58] Morales-Baquero R, Pulido-Villena E, Reche I (2006). Atmospheric inputs of phosphorus and nitrogen to the southwest Mediterranean region: Biogeochemical responses of high mountain lakes. Limnol. Oceanogr..

[59] Martínez R (1977). Phytoplankton species, biomass, and diversity in Lake La Caldera (S. Nevada). Act. Hydrobiol..

[60] APHA. Standard Methods for the Examination of Water and Wastewater. American Public Health Association (1992).

[61] Steeman-Nielsen E (1952). The use of radioactive carbon (^14^C) for measuring organic production in the sea. J. Cons. int.Explor. Mer..

[62] Sterner, R. W. & Elser, J. J. *Ecological Stoichiometry: The Biology of Elements from Molecules to Biosphere*. Princeton University Press, Princeton, New Jersey, USA (2002).

[63] *Statistica for Windows*. Release 7.1. Statsoft, Tulsa, OK (2005).

[64] Quinn, G. & Keough, M. *Experimental Design and Data Analysis for Biologists*. Cambridge University Press (2002).

