# Supplementary material for: Vulnerability of mixotrophic algae to nutrient pulses and UVR in an oligotrophic Southern and Northern Hemisphere lake
Source: Sci Rep. 2017 Jul 24;7:6333. doi: 10.1038/s41598-017-06279-9 (PMC5524960; doi:10.1038/s41598-017-06279-9)
Supplement: Supplementary file 1 — Supplementary Information [file 41598_2017_6279_MOESM1_ESM.doc]

Supporting information for

Vulnerability of mixotrophic algae to nutrient pulses and UVR in an oligotrophic Southern and Northern Hemisphere lake

P. Carrillo^1^*, J.M. Medina-Sánchez^2^, M.Villar-Argaiz^2^, F.J. Bullejos^3^, C. Durán^1^, M. Bastidas- Navarro^4^, M.S. Souza^4^ , E.G.Balseiro^4^, B.E. Modenutti^4^

* Corresponding author: P. Carrillo (email: pcl@ugr.es), Phone: + 34 958 241000 ext. 20002

**Table S1**. Chlorophyll *a* (Chl *a*; µg L^-1^), algal biomass (µg C L^-1^), and sestonic elemental ratios (C:P; C:N) in the mesocosms during the pre-experiment period (1 month) in lake La Caldera and in lake Los Cántaros,, +UVR: full sunlight; -UVR: screened sunlight (>400 nm). Mean values ± Standard Deviation. Numbers in bold indicate significant UVR effect (t-student, p < 0.05)

| Lakes  Variables | La Caldera | | | | | Los Cántaros | | | |
| --- | --- | --- | --- | --- | --- | --- | --- | --- | --- |
|  | +UVR | | -UVR | +UVR | | | -UVR | |  |
| Chl *a* | 1.44±0.18 | 2.52±0.89 | | | 0.48±0.28 | | | 0.50±0.18 | |
| Algal Biomass | 24.33±6.89 | 26.08±2.13 | | | 10.46±3.88 | | | 11.25±4.33 | |
| C:P | **97.39±31.02** | **409.7±156.77** | | | **787.77 ±36.4** | | | **1009.7±116.7** | |
| C:N | **3.01±2.42** | **9.81±2.81** | | | 15.15±2.31 | | | 15.45±0.81 | |

|  |  |  |  |  |  |  |  |  |  |  |
| --- | --- | --- | --- | --- | --- | --- | --- | --- | --- | --- |

**Table S2.** Results of the two-way split-plot analysis of variance of UVR and nutrient effects on phytoplanktonic biomass (PB), chlorophyll *a* (Chl *a*), sestonic C content, sestonic N content, sestonic P, P cell quota, C:P ratio. Numbers in bold indicate significant interactive effect between two factors.

|  |  |  |  |  |  |  |  |  |  |  |  |  |  |  |  |  |  |  |  |  |  |  |  |
| --- | --- | --- | --- | --- | --- | --- | --- | --- | --- | --- | --- | --- | --- | --- | --- | --- | --- | --- | --- | --- | --- | --- | --- |
|  |  | PB | |  | Chl *a* | |  | Sestonic C | |  | Sestonic N | |  | Sestonic P | | P cell quota | |  | Chl *a*:C | |  | C:P ratio | |
| La Caldera lake | |  |  |  |  |  |  |  |  |  |  |  |  |  |  |  |  |  |  |  |  |  |  |
| Main plot effect | | F_1,4_ | *p* |  | F_1,4_ | *p* |  | F_1,4_ | *p* |  | F_1,4_ | *p* |  | F_1,4_ | *p* | F_1,4_ | *p* |  | F_1,4_ | *p* |  | F_1,4_ | *p* |
|  | UVR | 58.224 | **0.002** |  | 3.34 | 0.141 |  | 2.31 | 0.203 |  | 2.99 | 0.159 |  | 43.10 | **0.003** | 68.44 | **0.001** |  | 0.85 | 0.408 |  | 27.26 | **0.006** |
| Sub-plot effect | |  |  |  |  |  |  |  |  |  |  |  |  |  |  |  |  |  |  |  |  |  |  |
|  | Nutrient | 54.36 | **0.002** |  | 33.70 | **0.004** |  | 13.80 | **0.021** |  | 13.18 | **0.022** |  | 13.07 | **0.022** | 46.31 | **0.002** |  | 111.04 | **<0.001** |  | 9.92 | **0.035** |
|  | UVR×Nutrient | 20.79 | **0.010** |  | 25.66 | **0.007** |  | 30.48 | **0.005** |  | 17.58 | **0.014** |  | 8.25 | **0.045** | 11.88 | **0.026** |  | 54.29 | **0.002** |  | 0.74 | 0.439 |
| Los Cántaros lake | |  |  |  |  |  |  |  |  |  |  |  |  |  |  |  |  |  |  |  |  |  |  |
| Main plot effect | |  |  |  |  |  |  |  |  |  |  |  |  |  |  |  |  |  |  |  |  |  |  |
|  | UVR | 46.22 | **0.002** |  | 8.98 | **0.040** |  | 0.00 | 0.950 |  | 0.00 | 0.978 |  | 2.40 | 0.197 | 5.34 | 0.082 |  | 35.05 | **0.004** |  | 3.58 | 0.131 |
| Sub-plot effect | |  |  |  |  |  |  |  |  |  |  |  |  |  |  |  |  |  |  |  |  |  |  |
|  | Nutrient | 337.26 | **<0.001** |  | 29.94 | **0.005** |  | 0.02 | 0.890 |  | 17.79 | **0.014** |  | 218.68 | **0.000** | 21.07 | **0.010** |  | 129.25 | **<0.001** |  | 67.20 | **0.001** |
|  | UVR×Nutrient | 16.17 | **0.016** |  | 12.40 | **0.024** |  | 4.22 | 0.109 |  | 0.22 | 0.667 |  | 12.35 | **0.025** | 29.42 | **0.006** |  | 61.53 | **0.001** |  | 1.90 | 0.240 |
|  |  |  |  |  |  |  |  |  |  |  |  |  |  |  |  |  |  |  |  |  |  |  |  |
|  |  |  |  |  |  |  |  |  |  |  |  |  |  |  |  |  |  |  |  |  |  |  |  |

**Table S3.** Types of interactive effects (UVR×Nutrient) calculated from the magnitude and direction of the additive effect and interactive effect (in absolute terms) on algal variables. Control, UVR and Nutrient correspond to response variable value in the -UVR_NP-_,+UVR_NP-_,-UVR_NP+_ treatments, respectively. ‘Non-additive’ corresponds to the response-variable value in the +UVR_NP+_ treatment, and ‘Additive effect’ is the sum of control value plus the single UVR and Nutrient effects. A: antagonistic interaction; S: synergistic interaction; Sestonic P: sestonic phosphorus; P cell quota: phosphorus cell quota; PP: primary production; EOC: excretion of organic carbon; Chl *a:* chlorophyll *a*, Chl *a*:C: chlorophyll *a*:carbon ratio, spPP: specific cell productivity; PNUE: photosynthetic nutrient-use efficiency; PB: phytoplanktonic biomass. The sign of the types of interactive effects and calculations are based on Piggot *et al*., 2015 (see Supplementary text S5).

|  |  | |  | |  | |  | |  | |  | |  | |  | |  | |  | |  |
| --- | --- | --- | --- | --- | --- | --- | --- | --- | --- | --- | --- | --- | --- | --- | --- | --- | --- | --- | --- | --- | --- |
|  | Response variables | | Sestonic P | | P cell quota | | PP | | EOC | | Chl a | | Chl a:C | | spPP | | PNUE | | PB | |  |
| La Caldera lake | | |  | |  | |  | |  | |  | |  | |  | |  | |  | |  |
|  | Control | | 0.05 | | 0.08 | | 0.29 | | 0.10 | | 1.69 | | 0.005 | | 0.015 | | 0.19 | | 24.12 | |  |
|  | UVR | | 0.08 | | 0.15 | | 0.25 | | 0.44 | | 0.41 | | 0.002 | | 0.015 | | 0.12 | | 26.57 | |  |
|  | Nutrient | | 0.07 | | 0.15 | | 0.14 | | 0.12 | | 3.25 | | 0.01 | | 0.01 | | 0.07 | | 28.58 | |  |
|  | Additive effect | | 0.10 | | 0.22 | | 0.09 | | 0.46 | | 1.97 | | 0.003 | | 0.010 | | -0.01 | | 2.65 | |  |
|  | Non-additive effect | | 0.23 | | 0.33 | | 5.28 | | 0.91 | | 5.97 | | 0.011 | | 0.245 | | 0.75 | | 35.18 | |  |
|  | Interactive effect | | **S+** | | **S+** | | **S+** | | **S+** | | **S+** | | **S+** | | **S+** | | **S+** | | **S+** | |  |
|  |  | |  | |  | |  | |  | |  | |  | |  | |  | |  | |  |
| Los Cantaros lake | |  | |  | |  | |  | |  | |  | |  | |  | |  | |  | |
|  | UVR | | 0.03 | | 2.98 | | 0.27 | | 2.73 | | -0.14 | | 0.002 | | 0.98 | | 0.35 | | 8.51 | |  |
|  | Nutrient | | 0.06 | | 2.84 | | 1.11 | | 2.66 | | 1.60 | | 0.01 | | 1.72 | | 0.61 | | 41.76 | |  |
|  | Additive effect | | 0.06 | | 2.70 | | 0.97 | | 2.79 | | 1.43 | | 0.007 | | 1.67 | | 0.49 | | 41.63 | |  |
|  | Non-additive effect | | 0.09 | | 7.30 | | 0.32 | | 2.12 | | 0.56 | | 0.002 | | 1.12 | | 0.14 | | 29.73 | |  |
|  | Interactive effect | | **S+** | | **S+** | | **A+** | | **S-** | | **A+** | | **A+** | | **A+** | | **S-** | | **A+** | |  |

**Table S4.** Results of the two-way split-plot analysis of variance of UVR and nutrient effects on primary production (PP), excreted organic carbon (EOC), percentage excreted organic carbon (%EOC), specific cell productivity (spPP), and photosynthetic nutrient-use efficiency (PNUE). Numbers in bold indicate significant interactive effect between the factors.

|  |  |  |  |  |  |  |  |  |  |  |  |  |  |  |  |
| --- | --- | --- | --- | --- | --- | --- | --- | --- | --- | --- | --- | --- | --- | --- | --- |
|  |  |  |  |  |  |  |  |  |  |  |  |  |  |  |  |
|  |  | PP | |  | EOC | |  | %EOC | |  | spPP | |  | PNUE | |
| La Caldera lake |  | F_1,4_ | *p* |  | F_1,4_ | *p* |  | F_1,4_ | *p* |  | F_1,4_ | *p* |  | F_1,4_ | *p* |
| Main plot effect | |  |  |  |  |  |  |  |  |  |  |  |  |  |  |
|  | UVR | 225.98 | **0.000** |  | 80.01 | **0.001** |  | 15.72 | **0.017** |  | 224.72 | **0.000** |  | 1936.17 | **0.000** |
| Sub-plot effect | |  |  |  |  |  |  |  |  |  |  |  |  |  |  |
|  | Nutrient | 222.62 | **0.000** |  | 3.89 | 0.120 |  | 0.71 | 0.446 |  | 226.33 | **0.000** |  | 62.42 | **0.001** |
|  | UVR×Nutrient | 240.31 | **0.000** |  | 2.77 | 0.171 |  | 6.44 | 0.064 |  | 247.20 | **0.000** |  | 138.16 | **0.000** |
| Los Cantaros lake |  |  |  |  |  |  |  |  |  |  |  |  |  |  |  |
| Main plot effect | |  |  |  |  |  |  |  |  |  |  |  |  |  |  |
|  | UVR | 24.61 | **0.008** |  | 5.69 | 0.076 |  | 133.81 | **0.000** |  | 32.77 | **0.004** |  | 48.34 | **0.002** |
| Sub-plot effect | |  |  |  |  |  |  |  |  |  |  |  |  |  |  |
|  | Nutrient | 8.68 | **0.042** |  | 16.06 | 0.016 |  | 17.81 | **0.013** |  | 5.99 | 0.071 |  | 1.51 | 0.287 |
|  | UVR×Nutrient | 11.86 | **0.026** |  | 22.98 | **0.009** |  | 63.49 | **0.001** |  | 2.69 | 0.176 |  | 32.30 | **0.005** |
|  |  |  |  |  |  |  |  |  |  |  |  |  |  |  |  |
|  |  |  |  |  |  |  |  |  |  |  |  |  |  |  |  |

**Figure S1.** Illustration of the experimental design used in lakes Los Cántaros and La Caldera. Field mesocosms are represented by cylinders and the grey color denotes exclusion of UVR. After one-month of incubation, water from each mesocosm was used to fill the microcosms which were either unamended or received nutrients to complete the four (2×2) treatments. After seven days, all response variables were measured.

**
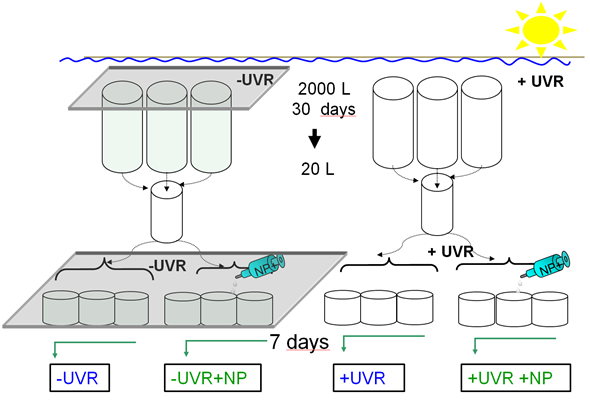
**

**Figure S2**. Chlorophyll *a* content (A, B) and Chl *a*:C ratio (C, D) under full sunlight (+UVR) and photosynthetically active radiation (-UVR) in non-enriched (NP-) and nutrient-enriched (NP_+_) treatments in La Caldera and Los Cántaros lakes. Data are expressed as mean values ± SD (n=3). Significant differences among treatments are denoted by different lower-case letters.

**Figure S3***.* Bacterivory rate (measured as sCBP, *see* Supplementary text S5) under full sunlight (+UVR) and photosynthetically active radiation (-UVR) in non-enriched (NP-) and nutrient-enriched (NP+) treatments in Los Cántaros lakes. Data are expressed as mean values ± SD (n=3). Significant differences among treatments are denoted by different lower-case letters.

.

**Supplementary text S1**

***Model ecosystems.*** The lake La Caldera has a surface area of approximately 2 ha and maximum depth of 14 m. It is usually ice-covered from October to June-July. As in most high-mountain lakes above the tree line, the water is highly transparent, with Secchi disk visibility reaching maximum depth (14 m) and low concentration of dissolved organic carbon (DOC < 1 mg C L^-1^) that allows a considerable intensity of UV radiation to penetrate deep into the lake^1,2^ , see the Results section). DIN:TP (by mass) ratio values are > 40^3^, characteristic of a strong P limitation throughout the ice-free period^3,4^ . This ecosystem undergoes inputs of nutrient-rich Saharan dust^5, 6^. Fish are absent and calanoid copepod *Mixodiaptomus laciniatus* is the dominant crustacean zooplankton in the pelagic zone. Los Cántaros is a mountain lake located in the Andes mountain range in north-eastern Patagonia, Argentina, 41º 00’ S, 71º 49’ W) in a glacial cirque at 1000 m. a.s.l. surrounded by forest of *Nothofagus*. The lake has a surface area of nearly 22 ha and maximum depth of 14 m. Mean chlorophyll *a* (Chl *a*) is < 1 µg L^-1^ and DOC < 0.7 mg L^-1 7^. The water is highly transparent (>10% of photosynthetically active radiation [PAR, 400–700 nm] penetrate to maximum depth) and, like other mountain lakes, receives a high UVR intensity^7,8^. The total phosphorous (TP) concentration is less than 10 µg L^-1^, this being characteristic of their oligotrophic conditions, and the atmospheric P loads are negligible^5^. Zooplankton is dominated by the calanoid copepod *Boeckella gibbosa*^8^. Fish are absent since this lake is isolated from the Nahuel Huapi systems by a waterfall 100 m high, which constitutes a natural barrier for upstream fish movement.

**Supplementary text S2**

It has been shown that AI is well correlated to the amount of TP linked to dry atmospheric deposition collected at a station near La Caldera^6^, and previous work in this lake has emphasized that atmospheric depositions constitute readily available nutrient sources for plankton^5,9^. We calculated the sum of daily events with AI > 1 (which represents a heavy deposition event) for the ice-free period considered for each lake and year. UV irradiance 324 nm represents an intermediate value between the available UVA and UVB wavelengths (305, 310, 324-5, and 380 nm). UV irradiance values before 2005 over La Caldera were obtained from the Total Ozone Mapping Spectrometer-Aerosol Index (TOMS-AI) developed by the Ozone Processing Team (National Aeronautics and Space Administration – Goddard Space Flight Center) ([http://ozoneaq.gsfc.nasa.gov](http://ozoneaq.gsfc.nasa.gov/))

**Supplementary text S3**

In La Caldera, Plexiglas UF3, a long-wave–pass plastic that transmits 85% of PAR but blocks UVR (< 380 nm), was used to cut off UVR, covering the enclosures and extending 2 m beyond the setup (Fig. S1). Further, the rack (subset) containing -UVR mesocosms was surrounded by several 2-m^2^ layers of Plexiglas UF3 to prevent any refractory solar UV radiation on the experimental area. In Los Cántaros, a similar UVR cut-off was reached with polyethylene for which the optical features were checked before the experiment using a double-beam spectrophotometer (Shimadzu UV2450, Shimadzu, Japan): cut-off at 380 nm and 85% transmittance above 400 nm. Finally, the top of each enclosure was covered with polyethylene for +UVR and Plexiglas UF3 (or the special polyethylene used in Los Cántaros) for -UVR to avoid external nutrient inputs during the incubation period but allow air exchange.

**Supplementary text S4**

An aliquot of variable volume between 50-100 mL from each sample was settled in an Utermöhl chamber of 2.6 cm diameter for 48 h to ensure complete sedimentation of the smallest phytoplankton species. Cells were counted in 100 randomly selected fields of view at 400x and 2000x magnification for ciliates and HNF or phytoplankton, respectively, under an inverted microscope (Leitz Fluovert FS, Leica, Wetzlar, Germany and Olympus IX70, Tokyo, Japan). Phytoplankton biomass was estimated by approximating the cell volume to their geometric shape and transforming it to carbon (C) units following Rocha and Duncan^10^.

**Supplementary text S5**

For bacterivory measurements, we followed basically the approach described by Medina-Sánchez *et al.*^11^. Briefly, a set of 4 (3 plus 1 blank) acid-cleaned and sterilized flasks per treatment, each filled with 25 ml of lake water with added [methyl-3H] thymidine (TdR; SA: 2.6–3.2 TBq mmol^-1^, Amersham Pharmacia; 15 nmol, saturating final concentration), was incubated in situ for a maximum of 90 min at the same depths as those for the PP incubations. This incubation time minimizes the potential artefact associated with label release and/or recycling^12^. After the incubations, the samples were preserved with neutral formaldehyde (3.4% final concentration), which was added to killed-control flasks before the incubations to quantify any passive incorporation or adsorption of the TdR tracer by the sestonic fraction. After incubations, the water from each flask was filtered by gravity through a 3-um pore-size cellulose-nitrate filter (Whatman), which was twice rinsed with 2 ml of 0.2-um filtered lake water, inserted into 2-mL sterile polypropylene micro-centrifuge vials and dissolved in acetone 90% to transfer the filtrated cells to the liquid phase. In this way, we quantified the incorporation of thymidine as bacterial production consumed by algal fraction; the <3-um filtrate served to quantify the remnant bacterial production, i.e. not consumed by bacterivores. The bacterial production for each fraction was quantified following the cold-TCA extraction and centrifugation protocol described by Smith and Azam^13^. The TdR incorporation rate was converted to equivalent bacterial cell number by using the conversion factor of 1x10^6^ cells mol TdR^-1^. The Bacterivory was estimated from this conversion factor and the abundance of bacterivores for each treatment as the specific consumption of bacterial production (sCBP, bacteria consumed cell^-1^ h^-1^).

**Supplementary references**

1. Carrillo, P., Medina-Sánchez J.M. & Villar-Argaiz, M. The interaction of phytoplankton and bacteria in a high-mountain lake: importance of the spectral composition of solar radiation, *Limnol. Oceanogr.* **47,** 1294–1306 (2002)

2. Carrillo, P., Delgado-Molina, J.A., Medina-Sánchez, J.M., Bullejos, F.J. & Villar-Argaiz, M. Phosphorus inputs unmask negative effects of ultraviolet radiation on algae in a high mountain lake. *Glob. Chang. Biol.* **14,** 423–439 (2008)

3. Villar-Argaiz, M., Medina-Sánchez, J.M., Cruz-Pizarro, L. & Carrillo, P. Inter and intra-annual variability in the phytoplankton community of a high mountain lake: the influence of external (atmospheric) and internal (recycled) sources of phosphorus. *Freshwater Biol.* **46,** 1017–1034 (2001)

4 . Carrillo, P., Reche, I., & Cruz-Pizarro, L. Intraspecific stoichiometric variability and the ratio of nitrogen to phosphorus resupplied by zooplankton. Freshwater Biol. 36, 363–374 (1996)

5. Mladenov, N. et al. Dust inputs and bacteria influence dissolved organic matter in clear alpine lakes. *Nat. Commun.* **2,** 10.1038/ncomms1411 (2011)

6. Morales-Baquero, R., Pulido-Villena, E. & Reche, I. Atmospheric inputs of phosphorus and nitrogen to the southwest Mediterranean region: Biogeochemical responses of high mountain lakes. *Limnol. Oceanogr.* **51**, 830-837 (2006)

7. Morris, D et al. The attenuation of solar UV radiation in lakes and the role of dissolved organic carbon. *Limnol. Oceanogr.* **40**, 1381-1391 (1995)

8. Souza, M.S. et al. Stoichiometric dietary constraints influence the response of copepods to ultraviolet radiation-induced oxidative stress, Limnol. Oceanogr. 53, 1024–1032 (2010)

9. Villar-Argaiz, M., Medina-Sánchez, J.M. & Carrillo, P. Microbial plankton response to contrasting climatic conditions: insights from community structure, productivity and fraction stoichiometry. *Aquat. Microb. Ecol.* **29**, 253–266 (2002)

10. Rocha, O. & Duncan, A. The relationship between cell carbon and cell volume in freshwater algal species used in zooplanktonic studies. J. Plankton Res. 7, 279–294 (1985)

11. Medina-Sánchez, J.M., Villar-Argaiz M.& Carrillo, P. Neither with nor without you: a complex algal control on bacterioplankton in a high mountain lake. Limnol. Oceanogr. 49,1722–1733 (2004)

12. Caron, D.A., Lessard,E.J., Voytek, M. & Dennett ,M.R.) Use of tritiated thymidine (TdR) to

estimate rates of bacterivory: implication of label retention and release by bacterivores. Mar. Microb. Food Webs, 7, 177-196 (1993)

13. Smith D.C., & Azam, F. A. simple, economical method for measuring bacterialprotein synthesis rates in seawater using 3H-leucine. Mar Microb Food Webs 6, 107–114 (1992)
